# Lipoprotein(a) Serum Levels in Diabetic Patients with Retinopathy

**DOI:** 10.1155/2013/943505

**Published:** 2013-06-05

**Authors:** Giulia Malaguarnera, Caterina Gagliano, Claudio Bucolo, Marco Vacante, Salvatore Salomone, Michele Malaguarnera, Daniela Giovanna Leonardi, Massimo Motta, Filippo Drago, Teresio Avitabile

**Affiliations:** ^1^International Ph. D. Program in Neuropharmacology, University of Catania, 95123 Catania, Italy; ^2^Department of Ophthalmology, University of Catania, 95123 Catania, Italy; ^3^Research Center “La Grande Senescenza”, University of Catania, 95125 Catania, Italy

## Abstract

*Background*. Atherogenic lipoproteins, such as total cholesterol, LDL cholesterol, oxidized low density lipoprotein, and triglycerides, are associated with progression of retinopathy. *Aim*. To evaluate the relationship between lipoprotein(a) and retinopathy in patients with type 2 diabetes mellitus. *Materials and Methods*. We enrolled 145 diabetic consecutive patients (82 females, 63 males; mean age 66.8 ± 12 years, mean duration of diabetes 9.4 ± 6.8 years). Presence and severity of retinopathy were evaluated. Serum lipid profile, including Lp(a) level, was assessed. *Results*. High Lp(a) levels have been observed in 54 (78.3%) subjects and normal levels in 13 (18.85%) subjects as regards diabetic patients with retinopathy. Lp(a) levels were high in 15 subjects (21.75%) and normal in 63 subjects (91.35%) as regards patients without retinopathy. *Conclusions*. Lp(a) levels are increased in a significant percentage of patients with retinopathy compared to diabetic patients without retinopathy. The impact of Lp(a) levels on diabetic retinopathy needs to be further investigated.

## 1. Introduction

In developed countries, diabetic retinopathy (DR) is the leading cause of vision loss in adults of working age [1]. The worldwide prevalence of diabetes was estimated to be 2.8% in 2000 and 4.4% in 2030 [2]. The prevalence rates of DR ranged from 7% to 12%. Kempen et al. reported that approximately 4.1 million US adults 40 years and older have diabetic retinopathy [3]. Early identification and treatment are key priorities for reducing the morbidity of diabetic eye disease. Population and family studies have shown the pathogenesis of DR due to the interaction of several environmental, nutritional, and genetic risk factors [4]. The pathophysiology of DR is not completely understood at this time, but various biochemical pathways have been implicated, including polyol accumulation (via the enzyme aldose reductase), accumulation of advanced glycation end products, formation of reactive oxygen species, and activation of protein kinase C (PKC) [5, 6]. Risk factors for DR include hyperglycemia, hypertension, and dyslipidemia. It has been demonstrated that atherogenic lipoproteins, such as total cholesterol, LDL cholesterol, oxidized low density lipoprotein, and triglycerides, are associated with progression of retinopathy, proliferative retinopathy, and the development of macular oedema [7–9]. Lp(a) is a LDL like molecule consisting of an apolipoprotein B100 particle attached by a disulphide bridge to apo(a). Lp(a) plasma concentrations are highly heritable and mainly controlled by the apo(a) gene located on chromosome 6q26-27 [10]. The kringles of apo(a) are to a great extent a homologue of the kringle IV of plasminogen. Lp(a) competes with plasminogen in binding to fibrin and plasminogen receptors on the surfaces of monocytes and vascular endothelial cells, which may attenuate the generation of plasmin, and reduces fibrinolytic activity in blood circulation [11, 12]. Many prospective epidemiological studies have reported positive associations of Lp(a) concentration with cardiovascular diseases [13]. However, there are few and conflicting studies examining the relationship between lipoprotein(a) and retinopathy in patients with type 2 diabetes mellitus (DM).

## 2. Methods and Materials

### 2.1. Study Design

This study was carried out on patients with type 2 DM regularly attending our outpatients clinic at Cannizzaro Hospital in Catania. We enrolled 145 diabetic consecutive patients (82 females, 63 males; mean age 66.8 ± 12 years, mean duration of diabetes 9.4 ± 6.8 years). The exclusion criteria were (1) patients who were already on lipid lowering drugs or glitazones; (2) females taking oral contraceptive pills or hormone replacement therapy; (3) familial hypercholesterolemia; (4) hypothyroidism; (5) patients with chronic liver disease; (6) Patients with kidney disease. Assessment of DR was performed by ophthalmoscopy and/or biomicroscopy through dilated pupils by a retinal specialist, and fluorescein angiography was obtained when indicated. Examination of the retina was done through dilated pupils to determine the level of nonproliferative DR or proliferative DR. DR was graded as no retinopathy and minimum, moderate, or severe retinopathy as published elsewhere [14]. For the purpose of this study, the comparison between the groups of patients was performed according to the presence or absence of DR, regardless of its degrees of severity. All patients participating in this study gave their written informed consent.

### 2.2. Methods

Venous blood samples were drawn from patients, and all examinations were performed at 8.00 h after an overnight fast. The samples were allowed to clot, and serum was separated from the erythrocytes by centrifugation at 4°C and at 1500 ×g for 15 min. Total cholesterol, triglycerides, and fasting plasma glucose were enzymatically measured (Roche/Hitachi 912 analyzer; Roche Diagnostics, Switzerland). The fasting plasma glucose concentrations were assayed using the glucose-oxidase method with intra- and interassay CVs of 0.8% and 2.1%, respectively. High density lipoprotein cholesterol (HDL-c) was measured enzymatically in the supernatant after precipitation of apolipoprotein B-containing lipoproteins by phosphotungstate/MgCl_2_. Low-density lipoprotein (LDL) level was calculated by using Friedewald's formula [15]. Measurement of HbA1c was made by high-performance liquid chromatography (Menarini Diagnostics, Italy). Apolipoprotein A1 and B100 were measured by using nephelometric methods [16, 17]; Lp(a) was assayed by immunonephelometry (Olympus AU 640 Medican Watford UK). Coefficients of variance were 3% and 4.2% for intra- and interassay variability, respectively. Lp(a) normal range in serum is up to 30 mg/dL. Anthropometric measurements including weight, height, waist, and hip measurements were obtained using standardized techniques. Height was measured with a tape to the nearest centimetre. Subjects were requested to stand upright without shoes with their back against the wall, heels together, and eyes directed forward. Weight was measured with a traditional spring balance that was kept on a firm horizontal surface. Subjects were asked to wear light clothing, and weight was recorded to the nearest 0.5 Kg. Body mass index (BMI) was calculated by using the formula: weight (Kg/height (m^2^)). Waist circumference was measured by using a nonstretchable measuring tape. The subjects were asked to stand erect in a relaxed position with both feet together on a flat surface; one layer of clothing was accepted. Waist girth was measured as the smallest horizontal girth between the costal margins and the iliac crest at minimal respiration. Hip was taken as the greatest circumference (the widest protrusion of the hip) on both sides; measurements were made to the nearest centimetre. Waist-to-hip ratio was calculated by dividing the waist circumference (cm) by the hip circumference (cm). Examination of the retina was done through dilated pupils to determine the level of nonproliferative DR, proliferative DR, and macular edema by qualified ophthalmologists (T.A.; C.G.). The definitions were based on the International Classification of Diabetic Retinopathy.

## 3. Statistical Analysis

Data were reported as median or mean ± standard deviation for continuous variables and as proportions for categorical variables. Continuous variables were all analyzed by Student's *t*-test and for proportions *χ* ^2^ test. Multiple logistic regression analysis was used to obtain the association between various indices and DR. A *P* value ≤ 0.05 was considered statistically significant.

## 4. Results

In our study 67 out of the 145 enrolled patients had DR (Table 1). The patients with DR had a longer duration of diabetes (13.8 versus 5.4 years; *P* < 0.01) and higher glycated haemoglobin levels (10.2% versus 8.7%; *P* < 0.01) compared to subjects without DR. As regards diabetic management, 5% of patients were on diet alone; 64% were on oral antidiabetic drugs, for example, metformin and/or sulfonylurea; 13% were on metformin + insulin; 10% were on sulfonylurea + insulin; 8% were on insulin alone. Lp(a) levels were measured in all subjects. We observed high Lp(a) levels in 54 (78.3%) subjects and normal levels in 13 (18.85%) subjects as regards diabetic patients with retinopathy. Lp(a) levels were high in 15 subjects (21.75%) and normal in 63 subjects (91,35%) as regards patients without retinopathy (Table 2). 

## 5. Discussion

Various epidemiological studies suggested that increased plasma Lp(a) is a strong and independent risk factor for cardiovascular disease [18, 19]. The risk of developing cardiovascular disease is increased in diabetic individuals [20]. DR represents a common and severe complication of diabetes [21]; thus there is a need to implement effective strategies being able to prevent DR and to identify specific and early predictors. The etiology of DR is multifactorial, but reported risk factors include increased duration of diabetes mellitus, as well as severity of hypertension and hyperglycemia [22, 23]. In patients with type 2 diabetes the higher levels of total cholesterol, the LDL, and the Apo A1 can increase not only the cardiovascular disease but also the DR [24]. Circulating Lp(a) levels were increased in type 2 diabetic patients and positively correlated with blood concentrations of haemoglobin A1c. The higher glycated haemoglobin and the bed control of glycaemia may have important implications for the increased susceptibility to atherosclerosis. The majority of studies in type 2 diabetes found increased levels of Lp(a) in plasma with a few exception. Most studies reported that Lp(a) levels are unchanged in these patients [25–28]. Jenkins et al. [29] showed an increase of Lp(a) in type 2 diabetes with retinopathy. The potential causal relationship between lipoprotein(a) and retinopathy is still not clear. Elevated Lp(a) levels may play a causative role in DR by damaging the microcirculation. On the other hand, Rainwater et al. [30] have reported that Lp(a) concentrations were significantly lower in type 2 diabetes population compared to matched nondiabetic subjects. Since type 2 diabetes is characterized by hyperinsulinemia, this may contribute to a decrease in Lp(a) in these patients. Correlation between Lp(a) levels and macrovascular diseases was found in many studies in type 2 diabetes [31–34]. Beyond a role for Lp(a) in the macrovascular complication of diabetes, the existence of an association between elevated Lp(a) levels and microvascular diseases including DR and neuropathy has been studied. The existence of this relationship has been hypothesized based on the potential for Lp(a) to cause vessel damage through lipoprotein oxidation and on the potential antifibrinolytic and prothrombotic effects of Lp(a). Although some studies have shown a relationship between elevated Lp(a) levels and active retinopathy in patients with type 1 diabetes mellitus, a large prospective study failed to demonstrate this association [29, 33, 35, 36]. In our study we observed that Lp(a) levels increased in a significant percentage of patients with retinopathy compared to diabetic patients without retinopathy. Limitations of our study are the modest size of the patients and the absence of separate models for men and women, due to the small numbers. Larger studies using more defined populations are required to better understand the relationship between Lp(a) concentrations and retinopathy in patients with type 2 diabetes mellitus. Although some studies have shown a relationship between elevated Lp(a) levels and active retinopathy in patients with type 1 diabetes mellitus, a large prospective study failed to demonstrate this association [33, 35]. Improved control of systemic metabolic factors is associated with reduced risks of microvascular complications, including retinopathy, and these benefits may persist longer than the period of intensive control, a phenomenon termed “metabolic memory” [37]. Unfortunately, microvascular complications may progress despite metabolic control representing an unfavourable type of metabolic memory. The mechanisms of metabolic memory are poorly understood, but various genetic and epigenetic influences have been suggested. In a case-control study on young type 1 diabetes patients, Gazzaruso et al. [36] showed high Lp(a) levels in patients with proliferative retinopathy compared to patients with nonproliferative or background retinopathy. No relationship with neuropathy was observed in this study, consistent with a large prospective study of complications in patients with type 1 diabetes mellitus, that failed to demonstrate a positive relationship between Lp(a) concentrations and diabetic nephropathy; this study also did not identify a relationship between plasma Lp(a) levels and the development of DR. In 412 Korean patients with type 2 diabetes, higher serum Lp(a) levels have been found in subject with proliferative DR versus those with either no DR or with nonproliferative DR [38]. In conclusion, Lp(a) levels may be increased in patients with retinopathy. Lp(a) is involved in the development of atherothrombosis and activation of acute inflammation exerting a proatherogenic and hypofibrinolytic effect. The impact of Lp(a) levels on DR needs to be further investigated by prospective studies and the assessment of apo(a) polymorphism.

## Figures and Tables

**Table 1:** Demographic characteristics of the study population.

Patients with type 2 diabetes mellitus (*n* = 145)	
Female/male	82/63
Age (years)	66.8 ± 12.4
Smokers/no smokers	92/53
BMI (kg/m^2^)	26.4 ± 2.2
Waist circumference (cm)	94.8 ± 9.56
Hip circumference (cm)	96.8 ± 8.44
Waist-to-hip ratio	0.98 ± 0.05
Systolic blood pressure (mmHg)	145.4 ± 14.2
Diastolic blood pressure (mmHg)	84.6 ± 9.8

**Table 2:** Laboratory parameters of subjects included in the study.

Parameter	Patients with retinopathy (*n* = 67)	Patients without retinopathy (*n* = 78)	*P *
Cholesterol total (mmol/L)	6.24 ± 1.08	5.87 ± 0.96	*P* = 0.031
Cholesterol HDL (mmol/L)	1.44 ± 0.36	1.36 ± 0.30	*P* = 0.147
Cholesterol LDL (mmol/L)	4.19 ± 0.99	3.87 ± 0.95	*P* = 0.049
Triglycerides (mmol/L)	1.67 ± 0.76	1.48 ± 0.88	*P* = 0.170
Apolipoprotein A1 (g/L)	1.54 ± 0.18	1.47 ± 0.20	*P* = 0.029
Apolipoprotein B (g/L)	1.08 ± 0.21	1.06 ± 0.19	*P* = 0.548
Lipoprotein(a) (mg/dL)	56.4 ± 28.2	34.1 ± 12.4	*P* = 0.000
Fasting plasma glucose (mg/dL)	172 ± 26	135 ± 28	*P* = 0.000
HbA1c (%)	8.8 ± 0.6	7.2 ± 0.7	*P* = 0.000
